# Genome Sequence of *Bacillus* sp. Strain FJAT-14515

**DOI:** 10.1128/genomeA.01123-13

**Published:** 2014-01-23

**Authors:** Guohong Liu, Bo Liu, Weiqi Tang, Jianmei Che, Yingzhi Lin, Yujing Zhu, Mingxing Su, Jianyang Tang

**Affiliations:** a Agricultural Bio-resource Institute, Fujian Academy of Agricultural Sciences, Fuzhou, Fujian, China; b Fujian Agicultural and Forestry University, Fuzhou, Fujian, China

## Abstract

We report the draft genome sequence of *Bacillus* sp. strain FJAT-14515. The genome is 5.44 Mb in length. It covers 5,263 genes with an average length of 791 bp, has a G+C value of 37.06%, and contains 67 tRNAs, 31 small RNAs, and 5 rRNA loci.

## GENOME ANNOUNCEMENT

*Bacillus* sp. strain FJAT-14515 (16S rRNA GenBank accession number JX262264), a mesophile and endospore-forming bacterium, was isolated from a soil sample collected from Taiwan. It grows optimally at a 0% NaCl (range, 0 to 5%) concentration in nutrient agar (NA) at 30°C (10 to 40°C) and pH 7.0 (range, pH 6 to pH 9). The 16S rRNA similarities between FJAT-14515 and the closest strains, *Bacillus muralis* DSM 16288^T^ and *Bacillus simplex* DSM 30646^T^, were <98% through the EzTaxon-e database (http://eztaxon-e.ezbiocloud.net/) (1). Devereux et al. (2) and Fry et al. (3) have proposed that a similarity of <98% in a 16S rRNA sequence should be considered evidence for separate species. So, the genome of *Bacillus* sp. FJAT-14515 was sequenced with a view to determining whether it is a novel species of the genus *Bacillus*.

The complete genome sequence was determined by Illumina Solexa technology at the Beijing Genomics Institute (BGI) (Shenzhen, China). Assembly was performed using SOAP de novo v 2.04 (4).

The genome assembly of *Bacillus* sp. FJAT-14515 (G+C content of 37.06%) has approximately 98-fold coverage. It contains 28 scaffolds totaling 5,443,019 bp (largest, 2,476,485 bp, and smallest, 598 bp). The scaffolds consist of 43 contigs totaling 5,436,942 bp (largest, 793,370 bp, and smallest, 204 bp). *N*_50_ scaffold lengths of 793,370 bp and *N*_50_ contig lengths of 435,981 bp were obtained. All assembly data were deposited in the DDBJ/EMBL/GenBank nucleotide sequence database.

Coding sequences (CDS) were predicted using Glimmer 3.02 (5) and further annotated using Uniprot, NCBInr, COG, and KEGG through BLASTP. tRNAs, rRNAs, and small RNAs (sRNAs) were identified using tRNAscan-SE (6), RNAmmer (7), and Rfam (8), respectively.

The genome contains 5,263 CDS with an average length of 789 bp, which represent 76.5% of the whole genome. The results of annotation showed that only 837 genes (15.9%) did not match any known protein in the current public protein databases. Of the 5,263 genes, 2,735 and 2,812 encode proteins assigned into COG functional categories and KEGGs, respectively. Additionally, 67 tRNAs, 31 sRNAs, 5 rRNAs, and 7 clustered regularly interspaced short palindromic repeats (CRISPRs) were identified in the genome.

### Nucleotide sequence accession number.

This whole-genome shotgun project for *Bacillus* sp. strain FJAT-14515 has been deposited at DDBJ/EMBL/GenBank under the accession number AYSD00000000.
